# Why COVID-19 Symptomatic Patients Did Not Seek Healthcare Service at the Early Phase of the Pandemic in Bangladesh: Evidence From a Cross-Sectional Study

**DOI:** 10.7759/cureus.65145

**Published:** 2024-07-22

**Authors:** Md Hasanul Banna Siam, Md Mahbub Hasan, Mohammad Meshbahur Rahman, Rashawan Raziur Rouf, Mohammad Sorowar Hossain

**Affiliations:** 1 Emerging and Neglected Diseases, Biomedical Research Foundation, Dhaka, BGD; 2 Genetic Engineering and Biotechnology, University of Chittagong, Chittagong, BGD; 3 Digital Health and Informatics, Biomedical Research Foundation, Dhaka, BGD; 4 Environment and Life Sciences, Independent University, Dhaka, BGD

**Keywords:** health-seeking behavior, health risk communication, healthcare inequality, social stigma, covid-19

## Abstract

Objective

The health-seeking behavior (HSB) of patients during an outbreak is crucial in mitigating the spread of disease. Poor HSB can increase mortality and make contact tracing more difficult. In this study, we aimed to examine the status of HSB among Bangladeshi educated individuals during the early phase of the COVID-19 pandemic when infection was spreading quickly, and social distancing measures were tightened across the country.

Methods

We conducted a cross-sectional survey online among Bangladeshi individuals using a virtual snowball sampling method to capture suspected COVID-19 patients who did not undergo COVID-19 diagnostic testing. Descriptive and inferential analyses were performed with statistical significance defined as p<0.05.

Results

The study consisted of 390 participants with 44.9% having a bachelor's degree, followed by 25.9% with a master's or PhD degree. Commonly reported symptoms among the participants included fever (77.7%), cough (50.5%), headache (46.2%), body pain (36.4%), sore throat (35.6%), anosmia (31.3%), anorexia (13.8%), diarrhea (11.4%), and dyspnea (11.3%). The most common reasons for not taking the COVID-19 test were limited testing facilities (48%), the risk of infection from the test center (46%), fear of social stigma (19%), considering COVID-19 infection as innocuous (18%), and fear of forced quarantine (5%). In regression analysis, participants who lived in rural areas were found to be 2.5 times more likely to buy medications from nearby pharmacies. Males were more likely to self-medicate, with male participants being 3.2 times more likely than female participants to consider COVID-19 infection as harmless (AOR: 3.2, CI: 1.28-7.98). Smokers were more likely to seek help from government hotlines and to use drugs at home. Respondents with higher monthly income were less likely to fear forced quarantine (AOR: 0.27, CI: 0.4-2.02) but more likely to consider the risk of infection at the test center (AOR: 1.75, CI: 0.88-3.49).

Conclusion

Our study highlights that non-compliance with public health guidelines by educated people during an epidemic indicates a general lack of health literacy and distrust in the healthcare system. Along with improved infrastructure, efforts to enhance public health risk communication and health literacy are necessary to rebuild public trust in the healthcare service.

## Introduction

The emergence of the COVID-19 pandemic has resulted in a formidable strain on the global healthcare system. Developing nations, such as Bangladesh, have been disproportionately affected due to limited healthcare resources and high population density. On March 8, 2020, the country reported its first case of COVID-19, with over 300,000 additional cases recorded within the subsequent six-month period [1]. This time was characterized by overtaxed medical services, as well as widespread anxiety and panic among the populace, culminating in a unique challenge to the healthcare system [2]. The crisis also put people's confidence in the existing system and their willingness to seek medical assistance to the test. Health-seeking behavior (HSB), which refers to any actions taken by patients at risk of illness to obtain appropriate treatment, is typically driven by people's awareness, knowledge, and perceptions of various health issues and can significantly influence population health outcomes [3]. The HSB influences the population's health outcomes and is potentially influenced by the timing and the type of healthcare service used [4].

In Bangladesh, healthcare resources are concentrated in urban secondary and tertiary hospitals, whereas the majority of the population resides in rural areas [5]. According to the estimates of the Ministry of Health and Family Welfare, there are only 3.05 physicians and 1.07 nurses per 10,000 population [5]. When initially dealing with the COVID-19 pandemic, Bangladesh lacked the capacity and skilled workforce necessary to perform RT-PCR tests, resulting in an unwanted decline in mass testing for COVID-19 [6]. This decline was also fueled by a lack of testing centers, testing scams, low trust in the healthcare system, and the high cost of testing at private facilities [7]. While the majority of people in developed countries visit health clinics or family physicians when they have a health issue, in resource-limited settings such as Bangladesh, a variety of contextual factors and obstacles might influence the access to and use of such healthcare services [8].

The recent findings in HSB during the COVID-19 pandemic have revealed a high proportion of social stigma and fear of being marginalized, leading to a decrease in people's willingness to pursue medical care [9]. In Bangladesh, the fear translated into mass panic, racial abuse, family relationship breakdown, and graveyards refusing to bury COVID-19 patients [2,10]. In a major health hazard such as COVID-19, various social factors can abruptly alter people’s behavior toward receiving medical care and undergoing diagnostic tests. Study shows that poor HSB may significantly increase mortality [11]. Understanding the HSB is very critical for containing the disease spread, contact tracing, and minimizing the gap in public health risk communication. In our study, we aim to assess the HSB among the educated segment of Bangladeshi society, hypothesizing that this population is more educated and aware of health issues during the COVID-19 pandemic and therefore more likely to opt for COVID-19 testing and seek medical advice.

An earlier version of this article was posted on the Research Square preprint server on December 15, 2021.

## Materials and methods

Study design and participants

For this study, we conducted a cross-sectional online survey among suspected COVID-19 patients between June 22, 2020, and July 7, 2020, when a strict country-wide social distancing measure was imposed to contain the spread of the virus [1]. We used a virtual snowball sampling method, where invitations describing the nature and purpose of the survey were sent through Facebook with a self-guided hyperlink to our research electronic data capture (REDCap) server to access the survey form [12]. This non-probability sampling method enabled us to enroll a hidden population that became our study population. We extracted this hidden population from a large pool of potential participants (source population) from Bangladeshi Facebook users who were above 18 years of age. We calculated the sample size using the Raosoft online sample size calculator (http://www.raosoft.com/samplesize.html) by assuming a 95% confidence level, 5% margin of error, and 50% response distribution. Because the population size of Bangladeshi Facebook users in 2021 was 46 million, the minimum recommended sample size was 385 [13]. Facebook was chosen as the platform for our internet-based survey because it is accessible to a large number of people in Bangladesh. Moreover, it was the only viable platform to conduct this study during the COVID-19 restriction. We initially identified and recruited a small pool of participants within the known vicinity using direct communication, and we asked them to share the questionnaire via Facebook among friends and send the survey form link via messenger app to someone they knew who was experiencing COVID-19-like symptoms. Any entry that did not meet our strict inclusion criteria was discarded. Our inclusion criteria required participants to (i) be at least 18 years old, (ii) be a smartphone/feature phone user, (iii) have at least a high school degree, and (iv) have symptoms similar to COVID-19, but (v) they had not been tested.

Questionnaire

The questionnaire was developed by a team of multidisciplinary experts from public health, microbiology, epidemiology, medical doctors, and statisticians considering the prevailing practices of HSBs in Bangladesh. In a pilot study, the questionnaire was tested on 15 participants who were excluded from the final analysis. In the final version, socio-demographic variables included the following: age (years), sex, the highest level of education (12th grade/bachelor/masters/PhD), occupation, current residence(urban/semi-urban/rural), monthly family income in Bangladeshi Taka (BDT), and smoking (cigarette) habit. Participants were asked about underlying medical conditions or comorbidities. The questionnaire included the most common symptoms of COVID-19 reported in the literature including fever, cough, runny nose, sore throat, breathing difficulty, anosmia, hypogeusia, anorexia, myalgia, and headache [14]. Participants were asked why they did not opt for the COVID-19 diagnostic test despite having COVID-19-like symptoms, and the options to choose from included the following: lengthy testing process, insufficient sample collection booths, fear of being quarantined, fear of being socially stigmatized if tested positive, fear of being infected during sampling, and nothing would happen if infected. Self-reported patients were also asked what measures they took after having COVID-19 symptoms, and the options included the following: taking treatment at home, taking hospital treatment, consulting with a medical doctor in a private chamber physically, seeking advice from family or known medical doctor through telemedicine, consulting with the nearest drug seller, asking advice from call center set by the government, taking self-medication, and not asking any advice. Participants were also asked whether they took alternative medicines, maintained social distancing, or followed lockdown restrictions.

Statistical analyses

Completed data were managed using the REDCap electronic data capture tool (www.project-redcap.org) hosted at the Biomedical Research Foundation (BRF), Bangladesh. The data were analyzed using Statistical Product and Service Solutions (SPSS, version 25.0; IBM SPSS Statistics for Windows, Armonk, NY). Descriptive statistics including frequency and percentage were done to describe the demographic characteristics; the percentage was done to describe the symptomatic clinical profile and patterns of HSB of COVID-19 symptomatic individuals. Inferential statistics, including the binary logistic regression model, was used to describe the associations between socio-demographic variables and the HSB of the study sample. To investigate the risk factors associated with not pursuing the COVID-19 test, bivariate analysis was performed for each exposure variable (age, sex, dwelling, smoking, education, profession, and income) to assess its association with the outcome variable. Exposure variables having p<0.05 level of significance in bivariate analysis were retained to construct the final model of multivariable logistic regression. The adjusted odds ratio (AOR) was obtained by clustering variables at the individual level. AOR and confidence interval (CI) were presented with the p-value set at <0.05.

Ethical approval

Ethics approval and formal permission for data collection for this study were obtained from the Biomedical Research Foundation, Bangladesh (Ref. no: BRF/ERB/2020/003). Informed written consent was obtained from the participants (aged 18 years and above only). They were adequately informed of the study's nature and purpose, of the right to withdraw their data, and were assured of maintaining the confidentiality of the data being used in this research.

## Results

A total of 390 self-reported symptomatic patients were enrolled who did not opt for the COVID-19 diagnostic test. The majority (75.6%) of participants were between the ages of 18 and 30 years, and only 6.7% belonged to the age group of 40 years and above. More male (79.5%) and urban-dwelling residents (80.3%) participated in the survey. About 45% had a bachelor’s degree, followed by 25.9% of post-graduation degrees, such as master’s and PhD, and 29.2% with higher secondary degrees (up to 12 grade). About half of the participants (49.2%) were students, followed by service holders (31.8%), and businessmen (5.9%). Less than one-fifth (13.1%) were without jobs or retired or housewives. Interestingly, about 90% of the respondents reported that they do not smoke. Table 1 shows the detailed demographic data.

**Table 1:** Demographic characteristics of the participants

Variable	Frequency (n)	Percentage (%)
Age (years)		
18 to <30	295	75.6
30 to <40	69	17.7
40 and over	26	6.7
Sex		
Female	80	20.5
Male	310	79.5
Dwelling		
Urban	313	80.3
Rural	77	19.7
Education		
Up to 12 grade	114	29.2
Bachelor	175	44.9
Masters and PhD	101	25.9
Profession		
Jobless/housewife/retired	51	13.1
Student	192	49.2
Business	23	5.9
Service	124	31.8
Income (BDT)		
<15k	73	18.7
15k-25k	100	25.6
25k-50k	106	27.2
50k and above	111	28.5
Smoking (cigarette)		
Yes	42	10.8
No	348	89.2

**Figure 1:**
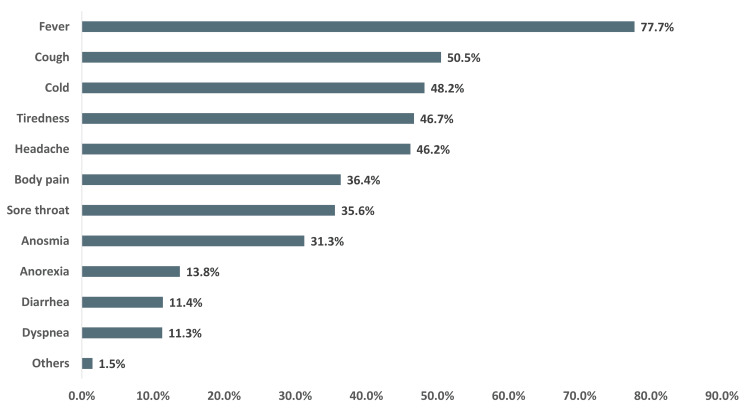
Symptomatic clinical profile of the participants

The symptomatic participants described fever as being the most common symptom (77.7%), followed by cough (50.5%), cold (48.2 %), tiredness (46.7%), headache (46.2%), body pain (36.4%), sore throat (35.6%), and anosmia or smell loss (31.3%). More than 60% of the participants reported that at least one of their family members had similar symptoms. Their source of information about common COVID-19 symptoms was Facebook (81.1%), newspapers (41.4%), other social media (41.9%), TV (36.3%), and physician or healthcare workers (20.5%) (Appendix Figure 4). A minority of participants reported anorexia (13.8%), diarrhea (11.4%), and breathing difficulty or dyspnea (11.3%). Figure 1 illustrates the symptomatic clinical profile of the participants.

**Figure 2:**
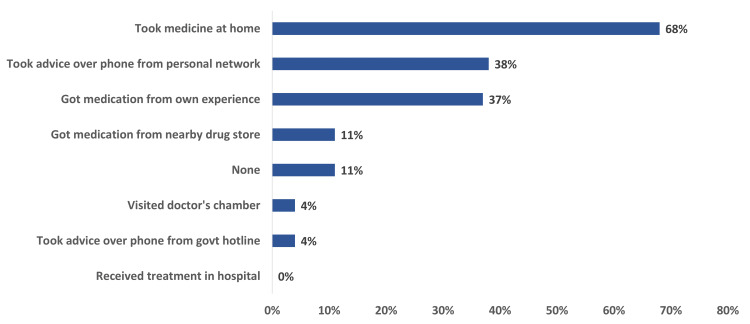
Pattern of medical care sought after having COVID-19-like symptoms

Most of the study participants took medication at home (68%); some reported taking advice from a personal network (38%), and others took medicines from personal experience (37%). About 11% of the respondents did not receive any type of medical care. Only 4% took advice over the phone from the government hotline number, and 4% visited a doctor’s chamber. Figure 2 illustrates the pattern of medical care sought after having COVID-19-like symptoms.

There was a significant association between the sex of the participants and taking advice over the phone from personal networks (p<0.007). The income range was also associated with taking advice over the phone (p<0.005). Self-medication from personal experience was significantly associated with sex (p<0.01), dwelling place (p<0.01), and income range (p<0.01). Age, type of profession, or educational qualification were not associated with any type of medical care pursued after having COVID-19-like symptoms (Appendix Table 4).

Table 2 shows the factors associated with seeking different types of medical care. Study participants who had the habit of smoking were more likely to seek advice over the phone from govt. hotline numbers (AOR: 8.78, CI: 1.98-38.89) and take medications at home (AOR: 2.20, CI: 0.98-4.95). Similar findings regarding taking medications at home were observed in the case of a profession for the service holders (AOR: 3.30 CI: 0.30-36.17) compared to those who did not have a job or were retired or homemakers. Males were more likely to visit doctor’s chambers (AOR: 3.02, CI: 0.36-25.12), as well as self-medicate (AOR: 1.92, CI: 1.04-3.5) than females. Participants from rural areas were 2.5 times more likely to purchase drugs from nearby stores (AOR: 2.54, CI: 1.15-5.62), and they had an 89% higher chance of practicing self-medication (AOR: 1.89, CI: 1.08-3.31) compared to the urban dwellers.

**Table 2:** Demographic factors associated with seeking different types of medical care

Variables		Take medication at home	Doctors’ chamber	Over-the-phone advice from a personal network	Over the phone from govt. hotline numbers	Nearby drug store	Got medications	Taken no medicine
		Adj. OR (95% CI)	Adj. OR (95% CI)	Adj. OR (95% CI)	Adj. OR (95% CI)	Adj. OR (95% CI)	Adj. OR (95% CI)	Adj. OR (95% CI)
Age (years)	18 to <30							
	30 to <40	1.80 (0.87-3.71)	0.25 (0.03-2.47)	1.41 (0.72-2.75)	0.85 (0.09-8.30)	0.59 (0.17-2.01)	0.76 (0.38-1.56)	1.01 (0.38-2.70)
	40 and over	0.87 (0.33-2.27)	1.46 (0.19-11.01)	1.48 (0.58-3.79)	0.10 (0.00-0.20)	0.36 (0.04-3.54)	1.89 (0.71-5.01)	0.54 (0.11-2.76)
Sex	Female							
	Male	0.74 (0.41-1.36)	3.02 (0.36-25.12)	0.56 (0.32-0.97)	0.23 (0.06-0.92)	0.49 (0.21-1.16)	1.92 (1.04-3.5)	1.08 (0.45-2.58)
Dwelling	Urban							
	Rural	0.49 (0.28-0.86)	1.74 (0.45-6.59)	0.58 (0.31-1.07)	0.31 (0.03-2.9)	2.54 (1.15-5.62)	1.89 (1.08-3.31)	1.16 (0.51-2.64)
Smoking (cigarette)	No							
	Yes	2.20 (0.98-4.95)	2.09 (0.51-8.50)	0.85 (0.41-1.78)	8.78 (1.98-38.89)	0.28 (0.06-1.32)	0.74 (0.36-1.52)	0.37 (0.08-1.67)
Education	Up to 12 grade							
	Bachelor	1.29 (0.75-2.23)	0.24 (0.06-1.05)	0.97 (0.56-1.68)	0.69 (0.19-2.49)	0.57 (0.27-1.23)	1.03 (0.60-1.78)	0.73 (0.33-1.62)
	Masters and PhD	1.36 (0.66-2.83)	0.84 (0.16-4.36)	0.93 (0.45-1.89)	0.01 (0.00-0.11)	0.36 (0.11-1.24)	0.60 (0.28-1.25)	0.63 (0.22-1.80)
Profession	Jobless/HW/retired							
	Student	1.43 (0.71-2.91)	1.58 (0.18-14.31)	2.29 (1.07-4.89)	1.83 (0.18-18.37)	2.81 (0.61-13.1)	1.16 (0.56-2.42)	0.53 (0.20-1.37)
	Business	0.67 (0.23-1.98)	1.56 (0.10-28.5)	2.51 (0.82-7.69)	0.20 (0.11-0.50)	7.53 (1.08-52.45)	0.46 (0.13-1.60)	1.49 (0.37-6.06)
	Service	0.91 (0.43-1.96)	1.8 (0.17-19.7)	2.04 (0.92-4.54)	3.30 (0.30-36.17)	5.41 (1.03-28.42)	2.57 (1.17-5.66)	0.81 (0.28-2.35)
Income (BDT)	<15k							
	15k-25k	1.09 (0.57-2.13)	0.80 (0.15-4.38)	0.51 (0.25-1.03)	1.59 (0.16-16.34)	1.03 (0.43-2.50)	1.43 (0.75-2.73)	0.46 (0.17-1.25)
	25k-50k	1.19 (0.59-2.37)	1.4 (0.26-7.54)	1.49 (0.76-2.9)	3.38 (0.36-31.46)	0.54 (0.19-1.52)	0.69 (0.34-1.38)	0.84 (0.33-2.12)
	50k and above	1.22 (0.59-2.49)	1.61 (0.28-9.35)	0.02 (0.00-0.31)	1.19 (0.10-14.17)	0.32 (0.10-1.08)	0.68 (0.32-1.38)	0.54 (0.19-1.52)
HW=Housewife/homemaker								

**Figure 3:**
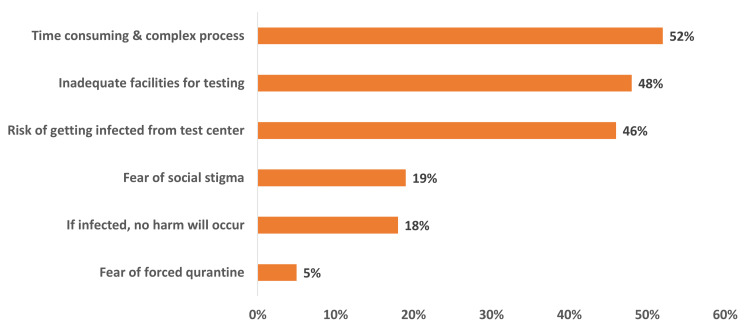
Reasons for not pursuing the COVID-19 diagnostic test

The most common reasons for not choosing to go through COVID-19 testing were that the process was perceived by the respondents as time-consuming and complex (52%), followed by inadequate facilities for testing (48%), risk of being infected from the test center (46%), fear of being socially stigmatized (19%), the consideration that COVID-19 will not cause any type of harm (18%), and being fearful of forced quarantine if they tested positive (5%). Figure 3 presents the reasons for not pursuing the COVID-19 test.

A significant difference was found between male and female participants regarding the consideration that COVID-19 infection will not cause any harm (p<0.005). A similar association was found for the income range (p<0.05) and those who live in an urban area (p<0.01). The type of profession was significantly associated with the fear of social stigma (p<0.01), the consideration that there is an inadequate facility for testing (p<0.01), and that the testing process is lengthy and complicated (p<0.03) (Appendix Table 5).

Table 3 shows the risk factors associated with not opting for the COVID-19 test. The odds of male participants considering COVID-19 infection as harmless was 3.2 times higher (AOR: 3.2, CI: 1.28-7.98) than the female participants. Participants with an age range of 40 and over were more likely to consider the fear of social stigma (AOR: 2.45, CI: 0.88-6.83), the complexity of the testing procedure (AOR: 2.71, CI: 1-7.35), and the fear of forced quarantine (AOR: 4.07, CI: 0.76-21.69) as impediments to opt for COVID-19 test. Respondents with an income of 50,000 BDT or above were less likely to fear forced quarantine (AOR: 0.27, CI: 0.4-2.02) but more likely to consider the risk of being infected from the test center (AOR: 1.75, CI: 0.88-3.49).

**Table 3:** Demographic risk factors associated with not pursuing the COVID-19 test

Variables		Time-consuming and complex process	Inadequate facilities for testing	Fear of social stigma	Fear of forced quarantine	Risk of getting infected from the test center	If infected nothing will happen
		Adj. OR (95% CI)	Adj. OR (95% CI)	Adj. OR (95% CI)	Adj. OR (95% CI)	Adj. OR (95% CI)	Adj. OR (95% CI)
Age (years)	18 to <30						
	30 to <40	1.69 (0.88-3.25)	0.79 (0.41-1.5)	0.59 (0.24-1.44)	0.92 (0.21-3.97)	1.05 (0.55-2.03)	1.31 (0.54-3.15)
	40 and over	2.71 (1-7.35)	1.08 (0.42-2.74)	2.45 (0.88-6.83)	4.07 (0.76-21.69)	0.77 (0.3-1.97)	0.4 (0.08-2)
Sex	Female						
	Male	0.83 (0.48-1.143)	1.81 (1.05-3.13)	1.42 (0.68-2.97)	1.1 (0.27-4.43)	0.73 (0.43-1.26)	3.2 (1.28-7.98)
Dwelling	Urban						
	Rural	0.99 (0.57-1.72)	0.64 (0.37-1.11)	1.16 (0.58-2.28)	1.54 (0.5-4.72)	0.47 (0.26-0.85)	1.79 (0.95-3.4)
Smoking (cigarette)	No						
	Yes	0.67 (0.34-1.33)	0.81 (0.41-1.59)	0.6 (0.21-1.64)	1.28 (0.32-5.13)	0.9 (0.45-1.78)	0.52 (0.2-1.37)
Education	Up to 12 grade						
	Bachelor	0.61 (0.34-1.33)	1.12 (0.67-1.87)	0.56 (0.28-1.08)	0.56 (0.19-1.71)	1.02 (0.6-1.7)	0.59 (0.31-11)
	Masters and PhD.	0.67 (0.34-1.31)	1.32 (0.68-2.64)	0.86 (0.38-1.98)	0.47 (0.1-2.17)	0.68 (0.34-1.35)	0.28 (0.11-0.73)
Profession	Jobless/HW/retired						
	Student	0.76 (0.39-1.48)	0.84 (0.43-1.63)	0.33 (0.16-1.89)	0.6 (0.13-2.8)	1.78 (0.88-3.57)	0.57 (0.24-1.33)
	Business	1.01 (0.35-2.89)	0.98 (0.35-2.77)	0.57 (0.17-1.89)	1.73 (0.26-11.39)	1.17 (0.39-3.49)	0.55 (0.14-2.21)
	Service	1.41 (0.68-2.91)	1.65 (0.8-3.41)	0.4 (0.17-0.93)	1.53 (0.31-7.56)	1.84 (0.87-3.89)	0.78 (0.3-2)
Income (BDT)	<15k						
	15k-25k	1.3 (0.69-2.46)	1.29 (0.68-2.45)	0.84 (0.38-1.85)	1.75 (0.45-6.77)	1.43 (0.74-2.76)	0.84 (0.4-1.8)
	25k-50k	1.56 (0.81-3)	1.39 (0.72-2.68)	0.94 (0.42-2.1)	1.17 (0.25-5.42)	1.54 (0.79-2.99)	0.54 (0.23-1.27)
	50k and above	1.01 (0.51-2.01)	0.85 (0.43-1.69)	0.63 (0.26-1.53)	0.27 (0.4-2.02)	1.75 (0.88-3.49)	1.15 (0.51-2.61)
HW=Housewife/homemaker							

## Discussion

In this study, we investigated the HSB of Bangladeshi individuals during the COVID-19 epidemic and described the reasons behind their reluctance to undergo COVID-19 testing despite being symptomatic and having free access to diagnostic services.

The majority of participants in this study were male, which was expected given that there are more male Facebook users (68.4%) in Bangladesh [15]. The higher percentage of male participants was also in line with the disproportionate case ratio observed in Bangladesh, where nearly 71% of COVID-19 cases and 77% of deaths occurred in the male population [1]. This is similar to what has been observed in studies conducted around the world [16]. One particular exception is the case of India, where males bore the higher burden of COVID-19 infection, but females recorded a significantly higher death rate [17]. Interestingly, male participants in this study were more confident about recovering from COVID-19 than female participants, which may result in neglect of personal safety guidelines. All symptomatic study participants were in the age range of 18-30, indicating the previous finding that young people were predominantly more affected by COVID-19 infection [1]. An initial study conducted in the UK showed that young men were more likely to show non-compliance with lockdown measures, which may contribute to a higher infection proportion [18]. However, it is important to note that participants in our study, despite being highly educated, did not comply with government-directed health guidelines. This trend observed among our study population through Facebook is likely to be manifested in a larger target population of educated Bangladeshis. Unlike other countries such as the USA, where multiple races and ethnicities are present, 98% of people in Bangladesh are Bengalis, and ethnic homogeneity plays a big role in culture, social behavior, and public opinion [19]. Moreover, Bangladesh has an overwhelmingly large number of young people (aged 18-30), and according to the Bangladesh Bureau of Statistics, there were 3.8 million and 2.4 million students enrolled in colleges and madrasahs in 2018, respectively [20]. There were also more than 800,000 students studying in 135 universities. A large pool of students and adults are very active on Facebook, and the number of new users is skyrocketing [21]. Similar to educated people, it is highly plausible that people with limited education may have more antagonistic behavior towards seeking health. Since health service utilization is directly influenced by health literacy, there is an urgent need to invest more in health literacy programs [22].

Self-medication was highly prevalent among the participants, and they received advice mostly from personal networks. By contrast, a smaller number of people visited doctors or used the government hotline telehealth service. Smokers, however, were found to be more likely to take medications at home and seek advice via the government telehealth service. This might be explained by media news about smokers being more susceptible to respiratory diseases and COVID-19. Among the most common medicines, ivermectin, azithromycin, and doxycycline were reported [23]. Self-medication was also more apparent in rural areas where people have a general lack of awareness about COVID-19. One study assessed that people in rural areas are better communicated with through direct discussion over the phone compared to text messages [24]. The authors also noted a higher proportion of women complying with the COVID-19 safety guidelines than men.

The most important findings of our study are the reasons why people refrained from COVID-19 diagnostic testing despite being symptomatic. The process was considered time-consuming and complex by the majority of the participants. One study reported that the average number of days between testing and receiving the result was five days in Sylhet and 10 days in Khulna [25]. The study also described the lack of skilled workforce to run the RT-PCR tests and the regional disparity in the availability of the testing center as major hurdles in increasing the test numbers. For instance, only 30 out of 64 districts had testing facilities as of June 30, 2020, whereas Dhaka city alone had 42 out of 68 operating testing centers [25]. This centralized healthcare facility was perhaps the second most common reason why participants in our study considered that there were inadequate facilities for testing. If the testing was done at a more improved rate, as one study reported, the number of positive cases would have increased by nearly 3.7 times [6].

Interestingly, people with higher incomes were less likely to fear forced quarantine than those with low- and middle incomes. This indicates a sharp disparity in the social hierarchy, as those with secure income or savings were better able to comply with stay-at-home orders or forced quarantine. This finding has important implications for lockdown measures and their effects on lower-income people. According to the Hrishipara Daily Financial Diaries project, low-income households suffered measurably during lockdowns, to the extent that their monthly income disappeared completely [26]. Families that were clients of microfinance providers (MFIs) found it difficult to obtain loans or withdraw savings since MFIs were shut down during the lockdown [27]. A study from BRAC revealed that people living in rural areas had considerably higher net income loss than their urban counterparts during the first phase of the pandemic [28].

Social stigma during the early phase of the pandemic also played a vital role in preventing people from testing for COVID-19. Social stigma is a common occurrence in epidemics, as observed during SARS and HIV [29]. However, the extent to which stigmatization presented itself during COVID-19 was notable because the infectious disease caused widespread panic. People chose not to test for COVID-19 because testing positive for it would force them to leave their rented apartment, stay away from family, or lose jobs [30]. Hatred, xenophobia, and denial of treatment were so widespread that hospitals rejected patients with flu, cough, or breathing difficulties [30]. Yellow journalism and social media influencers contributed to the infodemic and disinformation regarding COVID-19. More research should be conducted on how to prepare for future epidemics and make better use of public health risk communication.

Our study has a few limitations. Firstly, due to virtual snowball sampling through Facebook, our findings could be interpreted as context-specific and might not be generalizable to a broader population. Future studies are required to involve a more representative sampling technique to improve generalizability. Secondly, because of its online nature, the collected data were dependent upon the honesty and recall ability of the participants. Thirdly, since the survey was conducted within a limited period to capture the early scenario of the epidemic, we could not enroll a higher number of participants. Notably, the study was conducted within the unprecedented context in the history having nationwide lock-down measures in effect. Locating a group of hidden populations was particularly challenging because people were afraid to disclose their state of disease for fear of losing jobs, being thrown out of the rented apartment, and being stigmatized by society. However, given the paucity of research done in this area, we believe our work establishes a baseline for future studies to construct evidence-based guidelines to improve the HSB of patients during epidemics.

## Conclusions

Understanding how people seek medical help during epidemics is critical for preventing disease spread. Our study has revealed that, despite having higher education, the HSB of people can be in stark contrast to national guidelines if they lack trust in the healthcare system. People were hesitant to take the COVID-19 test or seek medical advice from government emergency numbers due to the lack of availability and complexity of the testing process, as well as fear of social stigma and the risk of becoming infected. The refusal of the educated class of society to comply with the public health guidelines infers a general lack of trust in the healthcare service during the pandemic.

## Figures and Tables

**Figure 4:**
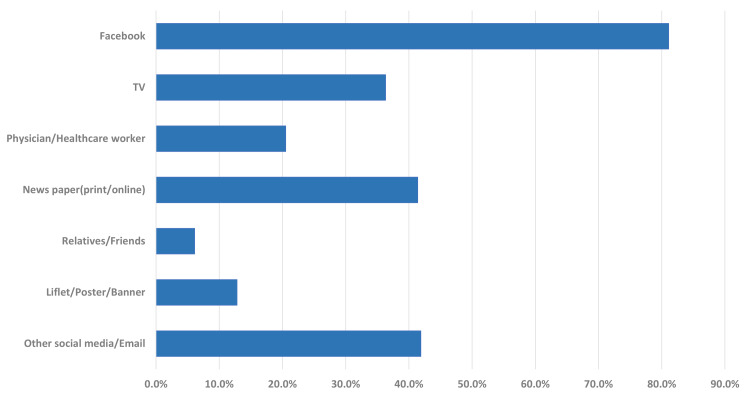
Participants' source of knowledge of COVID-19 symptoms

## Appendices

**Table 4:** Socio-demographic distribution and its association with respondents' HSB during COVID-19 HSB: Health-seeking behavior

Variables		n (%)	Taking medication at home			Doctors' chamber			Over-the-phone advice from a personal network			Over the phone from govt. hotlines			Nearby drug store			Got medication from own experience			Taken no medicine		
			Yes n(%)	No n(%)	p-value	Yes n(%)	No n(%)	p-value	Yes n(%)	No n(%)	p-value	Yes n(%)	No n(%)	p-value	Yes n(%)	No n(%)	p-value	Yes n(%)	No n(%)	p-value	Yes n(%)	No n(%)	p-value
Age (years)	18 to <30	295 (75.6)	197 (66.8)	98 (33.2)	0.205	12 (4.1)	283 (95.9)	0.34	106 (35.9)	189 (64.1)	0.264	14 (4.7)	281 (95.3)	0.252	37 (12.5)	258 (87.5)	0.133	112 (38)	183 (62)	0.426	33 (11.2)	262 (88.8)	0.85
	30 to <40	69 (17.7)	53 (76.8)	16 (23.2)		1 (1.4)	68 (98.6)		31 (44.9)	38 (55.1)		1 (1.4)	68 (98.6)		4 (5.8)	65 (94.2)		21 (30.4)	48 (69.6)		8 (11.6)	61 (88.4)	
	40 and over	26 (6.7)	16 (61.5)	10 (38.5)		2 (7.7)	24 (92.3)		12 (46.2)	14 (53.8)		0 (0)	26 (100)		1 (3.8)	25 (96.2)		11 (42.3)	15 (57.7)		2 (7.7)	24 (92.3)	
Sex	Female	80 (20.5)	60 (75)	20 (25)	0.140	1 (1.3)	79 (98.7)	0.18	41 (51.3)	39 (48.8)	0.007	6 (7.5)	74 (92.5)	0.06	10 (12.5)	70 (87.5)	0.58	19 (23.8)	61 (76.3)	0.01	8 (10)	72 (90)	0.74
	Male	310 (79.5)	206 (66.5)	104 (33.5)		14 (4.5)	296 (95.5)		108 (34.8)	202 (65.2)		9 (2.9)	301 (97.1)		32 (10.3)	278 (89.7)		125 (40.3)	185 (59.6)		35 (11.3)	275 (88.7)	
Dwelling	Urban	313 (80.3)	224 (71.6)	89 (28.4)	0.004	11 (3.5)	302 (96.5)	0.49	131 (41.9)	182 (58.1)	0.003	14 (4.5)	299 (95.5)	0.19	27 (8.6)	286 (91.4)	0.01	105 (33.5)	208 (66.5)	0.01	33 (10.5)	280 (89.5)	0.54
	Rural	77 (19.7)	42 (54.5)	35 (45.5		4 (5.2)	73 (94.8)		18 (23.4)	59 (76.6)		1 (1.3)	76 (98.7)		15 (19.5)	62 (80.5)		39 (50.6)	38 (49.4)		10 (13)	67 (87)	
Smoking (cigarette)	No	348 (89.2)	233 (67)	115 (33)	0.127	12 (3.4)	336 (96.6)	0.24	136 (39.1)	212 (60.9)	0.31	10 (2.9)	338 (97.1)	0.004	40 (11.5)	308 (88.5)	0.18	128 (36.8)	220 (63.2)	0.87	41 (11.8)	307 (88.2)	0.17
	Yes	42 (10.8)	33 (78.6)	9 (21.4)		3 (7.1)	39 (92.9)		13 (31)	29 (69)		5 (11.9)	37 (88.1)		2 (4.8)	40 (95.2)		16 (38.1)	26 (61.9)		2 (4.8)	40 (95.2)	
Education	Up to 12 grade	114 (29.2)	73 (64)	41 (36)	0.49	7 (6.1)	107 (93.9)	0.13	42 (36.8)	72 (63.2)	0.58	6 (5.3)	108 (94.7)	0.07	19 (16.7)	95 (83.7)	0.02	43 (37.7)	71 (62.3)	0.3	15 (13.2)	99 (86.8)	0.69
	Bachelor	175 (44.9)	121 (69.1)	54 (30.9)		3 (1.7)	172 (98.3)		64 (36.6)	111 (63.4)		9 (5.1)	166 (94.9)		18 (10.3)	157 (89.7)		70 (40)	105 (60)		18 (10.3)	157 (89.7)	
	Masters and PhD	101 (25.9)	72 (71.3)	29 (28.7)		5 (5)	96 (95)		43 (42.6)	58 (57.4)		0 (0)	101 (100)		5 (5)	96 (95)		31 (30.7)	70 (63.3)		10 (9.9)	91 (90.1)	
Profession	Jobless/housewife/retired	51 (13.1)	33 (64.7)	18 (35.3)	0.56	1 (2)	50 (98)	0.9	14 (27.5)	37 (72.5)	0.39	1 (2)	50 (98)	0.62	2 (3.9)	49 (96.1)	0.17	15 (29.4)	36 (70.6)	0.09	8 (15.7)	43 (84.3)	0.47
	Student	192 (49.2)	133 (69.3)	59 (30.7)		8 (4.2)	184 (95.8)		75 (39.1)	117 (60.9)		9 (4.7)	183 (95.3)		25 (13)	167 (87)		69 (35.9)	123 (64.1)		19 (9.9)	173 (90.1)	
	Business	23 (5.9)	13 (56.3)	10 (43.5)		1 (4.3)	22 (95.6)		9 (39.1)	14 (60.9)		0 (0)	23 (100)		4 (17.4)	19 (82.6)		5 (21.7)	18 (78.3)		4 (17.4)	19 (82.6)	
	Service	124 (31.8)	87 (70.2)	37 (29.8)		5 (4)	119 (96)		51 (41.1)	73 (58.9)		5 (4)	119 (96)		11 (8.9)	113 (91.1)		55 (44.4)	69 (55.6)		12 (0.96)	112 (90)	
Income (BDT)	<15k	73 (18.7)	44 (60.3)	29 (39.7)	0.3	3 (4.1)	70 (95.8)	0.95	24 (32.9)	49 (67.1)	0.005	1 (1.4)	72 (98.6)	0.29	12 (16.4)	61 (83.6)	0.01	31 (42.5)	42 (57.5)	0.01	12 (16.4)	61 (83.6)	0.2
	15k-25k	100 (25.6)	66 (66)	34 (34)		3 (3)	97 (97)		23 (23)	77 (77)		4 (4)	96 (96)		17 (18)	83 (83)		49 (49)	51 (51)		8 (8)	92 (92)	
	25k-50k	106 (27.2)	76 (71.7)	30 (28.3)		4 (3.8)	102 (98)		51 (48.1)	55 (51.9)		7 (6.6)	99 (93.4)		8 (7.5)	98 (92.5)		29 (27.4)	77 (71.6)		14 (13.2)	92 (86.8)	
	50k and above	111 (28.5)	80 (72.1)	31 (27.9)		5 (4.5)	106 (95.5)		51 (45.9)	60 (54.1)		3 (2.7)	108 (97.3)		5 (4.5)	106 (95.5)		35 (31.5)	76 (68.5)		9 (8.1)	102 (91.9)	

**Table 5:** Socio-demographic distribution and its association with the reasons for not undergoing the COVID-19 test

Variables		n (%)	Time-consuming and complex process			Inadequate facilities for testing			Fear of social stigma			Fear of forced quarantine			Risk of getting infected from the test center				If infected nothing will happen		
			Yes n(%)	No n(%)	p-value	Yes n(%)	No n(%)	p-value	Yes n(%)	No n(%)	p-value	Yes n(%)	No n(%)	p-value	Yes n(%)	No n(%)	p-value	Yes n(%)		No n(%)	p-value
Age (years)	18 to <30	295 (75.6)	139 (47.1)	156 (52.9)		137 (46.4)	158 (53.6)		55 (18.6)	240 (81.4)		14 (4.7)	281 (95.3)		136 (46.1)	159 (53.9)		58 (19.7)		237 (80.3)	
	30 to <40	69 (17.7%)	45 (65.2)	24 (34.8)	0.002	36 (52.2)	33 (47.8)	0.57	9 (13)	60 (87)	0.018	3 (4.3)	66 (95.7)	0.306	32 (46.4)	37 (53.6)	0.748	12 (17.4)		57 (82.6)	0.311
	40 and over	26 (6.7%)	19 (73.1)	7 (26.9)		14 (53.8)	12 (46.2)		10 (38.5)	16 (61.5)		3 (11.5)	23 (88.5)		10 (38.5)	16 (61.5)		2 (7.7)		24 (92.3)	
Sex	Female	80 (20.5)	45 (56.3)	35 (43.8)	0.39	30 (37.5)	50 (62.5)	0.36	13 (16.3)	67 (83.8)	0.49	3 (3.8)	77 (96.3)	0.53	44 (55)	36 (45)	0.06	6 (7.5)		74 (92.5)	0.005
	Male	310 (79.5)	158 (51)	152 (49)		157 (50.6)	153 (49.4)		61 (19.7)	249 (80.3)		17 (5.5)	293 (94.5)		134 (43.2)	176 (56.8)		66 (21.3)		244 (78.7)	
Dwelling	Urban	313 (80.3)	167 (53.4)	146 (46.6)	0.29	157 (50.2)	156 (49.8)	0.08	57 (18.2)	256 (81.8)	0.44	14 (4.5)	299 (95.5)	0.24	156 (49.8)	157 (50.2)	0.001	50 (16)		263 (84)	0.01
	Rural	77 (19.7)	36 (46.8)	41 (53.2)		30 (39)	47 (61)		17 (22.1)	60 (77.9)		6 (7.8)	71 (92.2)		22 (28.6)	55 (71.4)		22 (28.6)		55 (71.4)	
Smoking (cigarette)	No	348 (89.2)	185 (53.2)	163 (46.8)		167 (48)	181 (52)		69 (19.8)	279 (80.2)		17 (4.9)	331 (95.1)		160 (46)	188 (54)		66 (19)		282 (81)	
	Yes	42 (10.8)	18 (42.9)	24(57.1)	0.21	20 (47.6)	22 (52.4)	0.96	5 (11.9)	37 (88.1)	0.22	3 (7.1)	39 (92.9)	0.53	18 (42.9)	24 (57.1)	0.7	6 (14.3)		36 (85.7)	0.46
Education	Up to 12 grade	114 (29.2)	61 (53.5)	53(46.5)		48 (42.1)	66 (57.9)		27 (23.7)	87 (76.3)		8 (7)	106 (93)		53 (46.5)	61 (53.5)		29 (25.4)		85 (74.6)	
	Bachelor	175 (44.9)	81 (46.3)	94 (53.7)	0.07	82 (46.9)	93 (53.1)	0.1	26 (14.9)	149 (35.1)	0.15	8 (4.6)	167 (95.4)	0.54	83 (47.4)	92 (52.6)	0.63	32 (18.3)		143 (81.7)	0.23
	Masters and PhD	101 (25.9)	61 (60.4)	40 (39.6)		57 (56.4)	44 (43.6)		21 (20.8)	80 (79.2)		4 (4)	97 (96)		42 (41.6)	59 (58.4)		11 (10.9)		90 (89.1)	
Profession	Jobless/housewife/retired	51 (13.1)	28 (54.9)	23 (45.1)	0.03	23 (45.1)	28 (54.9)	0.01	18 (35.3)	33 (64.7)	0.01	3 (5.9)	48 (94.1)	0.27	17 (33.3)	34 (66.7)	0.16	11 (21.6)		40 (78.4)	0.83
	Student	192 (49.2)	86 (44.8)	106 (55.2)		79 (41.1)	113 (58.9)		31 (16.1)	161 (83.)		7 (3.6)	185 (96.4)		92 (47.9)	100 (52.1)		37 (19.3)		155 (80.7)	
	Business	23 (5.9)	13 (56.5)	10 (43.5)		11 (47.8)	12 (52.2)		6 (26.1)	17 (83.9)		3 (13)	20 (87)		8 (34.8)	15 (65.2)		4 (17.4)		19 (82.6)	
	Service	124 (31.8)	76 (61.3)	48 (38.7)		74 (59.9)	50 (40.3)		19 (15.3)	105 (84.7)		7 (5.6)	117 (94.4)		61 (49.2)	63 (50.8)		20 (16.1)		104 (83.9)	
Income (BDT)	<15k	73 (18.7)	32 (43.8)	41 (56.2)	0.35	30 (41.1)	43 (58.9)	0.44	18 (24.7)	55 (75.3)	0.46	4 (5.5)	69 (94.5)	0.13	25 (34.2)	48 (65.8)	0.14	20 (27.4)		53 (72.6)	0.05
	15k-25k	100 (25.6)	52 (52)	48 (48)		50 (50)	50 (50)		18 (21)	81 (82)		9 (9)	91 (91)		45 (45)	55 (55)		20 (20)		80 (80)	
	25k-50k	106 (27.2)	61 (57.5)	45 (42.5)		56 (52.8)	50 (47.2)		21 (19.8)	85 (80.2)		5 (4.7)	101 (95.3)		51 (48.1)	55 (51.9)		12 (11.3)		94 (88.7)	
	50k and above	111 (28.5)	58 (52.3)	53 (47.7)		51 (45.9)	60 (54.1)		17 (15.3)	94 (84.7)		2 (1.8)	109 (95.3)		57 (51.4)	54 (48.6)		20 (18)		91 (82)	

## References

[1] Siam MH, Hasan MM, Tashrif SM, Rahaman Khan MH, Raheem E, Hossain MS (2021). Insights into the first seven-months of COVID-19 pandemic in Bangladesh: lessons learned from a high-risk country. Heliyon.

[2] Hossain MS, Ferdous S, Siddiqee MH (2020). Mass panic during Covid-19 outbreak - a perspective from Bangladesh as a high-risk country. J Biomed Anal.

[3] Shaikh BT, Hatcher J (2005). Health seeking behaviour and health service utilization in Pakistan: challenging the policy makers. J Public Health (Oxf).

[4] Poortaghi S, Raiesifar A, Bozorgzad P, Golzari SE, Parvizy S, Rafii F (2015). Evolutionary concept analysis of health seeking behavior in nursing: a systematic review. BMC Health Serv Res.

[5] (2024). Health workforce. https://www.who.int/health-topics/health-workforce#tab=tab_1.

[6] Khan MHR, Howlader T (2020). Breaking the back of COVID-19: is Bangladesh doing enough testing?. J Biomed Anal.

[7] Cousins S (2020). Bangladesh's COVID-19 testing criticised. Lancet.

[8] Kok MC, Kane SS, Tulloch O (2015). How does context influence performance of community health workers in low- and middle-income countries? Evidence from the literature. Health Res Policy Syst.

[9] Villa S, Jaramillo E, Mangioni D, Bandera A, Gori A, Raviglione MC (2020). Stigma at the time of the COVID-19 pandemic. Clin Microbiol Infect.

[10] (2024). Coronavirus reshaping aspects of death; some even denied funerals. cited 6 Oct.

[11] Chileshe M, Mulenga D, Mfune RL (2020). Increased number of brought-in-dead cases with COVID-19: is it due to poor health-seeking behaviour among the Zambian population?. Pan Afr Med J.

[12] Baltar F, Brunet I (2012). Social research 2.0: virtual snowball sampling method using Facebook. Internet Res.

[13] (2024). Leading countries based on Facebook audience size as of April 2024. https://www.statista.com/statistics/268136/top-15-countries-based-on-number-of-facebook-users/.

[14] Li LQ, Huang T, Wang YQ (2020). COVID-19 patients' clinical characteristics, discharge rate, and fatality rate of meta-analysis. J Med Virol.

[15] Kemp S (2024). Digital 2021: Bangladesh. Digital.

[16] Dehingia N, Raj A (2021). Sex differences in COVID-19 case fatality: do we know enough?. Lancet Glob Health.

[17] Joe W, Kumar A, Rajpal S (2020). Equal risk, unequal burden? Gender differentials in COVID-19 mortality in India. J Glob Heal Sci.

[18] Levita L (2020). Initial research findings on the impact of COVID-19 on the well-being of young people aged 13 to 24 in the UK. COVID-19 Psychological Research Consortium.

[19] Caprio S, Daniels SR, Drewnowski A, Kaufman FR, Palinkas LA, Rosenbloom AL, Schwimmer JB (2008). Influence of race, ethnicity, and culture on childhood obesity: implications for prevention and treatment: a consensus statement of Shaping America's Health and the Obesity Society. Diabetes Care.

[20] Bangladesh Statistics 2019 (2024). Bangladesh - Statistics & facts. Dhaka.

[21] Facebook users increase by 10 million in Bangladesh (2024). Facebook users increase by 10 million in Bangladesh. cited 11 Jul.

[22] Das S, Mia MN, Hanifi SM, Hoque S, Bhuiya A (2017). Health literacy in a community with low levels of education: findings from Chakaria, a rural area of Bangladesh. BMC Public Health.

[23] Nasir M, Chowdhury ASMS, Zahan T (2020). Self-medication during COVID-19 outbreak: a cross sectional online survey in Dhaka city. Int J Basic Clin Pharmacol.

[24] Siddique A, Rahman T, Pakrashi D (2024). Raising health awareness in rural communities: a randomized experiment in Bangladesh and India. Rev Econ Stat.

[25] Rahaman KR, Mahmud MS, Mallick B (2020). Challenges of testing COVID-19 cases in Bangladesh. Int J Environ Res Public Health.

[26] (2024). Hrishipara Daily Financial Diaries - Corona virus. https://sites.google.com/site/hrishiparadailydiaries/home/corona-virus.

[27] (2024). Covid-19 and low-income households in central Bangladesh. cited 6 Oct.

[28] (2024). Rapid perception survey on COVID-19 awareness and economic impact. https://www.brac.net/images/news/2020/Perception-Survey-Covid19.pdf.

[29] Des Jarlais DC, Galea S, Tracy M, Tross S, Vlahov D (2006). Stigmatization of newly emerging infectious diseases: AIDS and SARS. Am J Public Health.

[30] (2024). Fear, hatred and stigmatization grip Bangladesh amid Covid-19 outbreak. https://www.tbsnews.net/thoughts/fear-hatred-and-stigmatization-grip-bangladesh-amid-covid-19-outbreak-61129.

